# Determination of Enantiomeric Excess by Optofluidic Microlaser near Exceptional Point

**DOI:** 10.1002/advs.202308362

**Published:** 2023-12-10

**Authors:** Zhiyang Xu, Yinzhou Yan, Xingyuan Wang, Xiaolei Wang, Zhixiang Zhou, Xi Yang, Tianrui Zhai

**Affiliations:** ^1^ Department of Physics and Optoelectronic Engineering Faculty of Science Beijing University of Technology Beijing 100124 China; ^2^ Institute of Laser Engineering Faculty of Materials and Manufacturing Beijing University of Technology Beijing 100124 China; ^3^ College of Mathematics and Physics Beijing University of Chemical Technology Beijing 100029 China; ^4^ Faculty of Environment and Life Beijing University of Technology Beijing 100124 China; ^5^ State Key Laboratory for Mesoscopic Physics and School of Physics Peking University Beijing 100871 China

**Keywords:** chirality discrimination, enantiomeric excess determination, exceptional point, optofluidic microlaser, whispering‐gallery mode

## Abstract

Enantiomeric excess (ee) is an essential indicator of chiral drug purification in the pharmaceutical industry. However, to date the ee determination of unknown concentration enantiomers generally involves two separate techniques for chirality and concentration measurement. Here, a whispering‐gallery mode (WGM) based optofluidic microlaser near exceptional point to achieve the ee determination under unknown concentration with a single technique is proposed. Exceptional point induces the unidirectional WGM lasing, providing the optofluidic microlaser with the novel capability to measure chirality by polarization, in addition to wavelength‐based concentration detection. The dual‐parameters detection of optofluidic microlaser empowers it to achieve ee determination of various unknown enantiomers without additional concentration measurements, a feat that is challenging to accomplish with other methods. Featuring the sensitivity enhancement and miniature structure of the WGM sensors, the obtained chiroptical response of the present approach is ≈30‐fold higher than that of the conventional optical rotation‐based polarimeter, and the reagent consumption is reduced by three orders of magnitude.

## Graphical abstract

By inducing the exceptional point, the whispering‐gallery mode‐based optofluidic microlaser is enhanced with the novel capability to measure chirality of enantiomer by polarization, in addition to wavelength‐based concentration detection. The dual‐parameters detection of optofluidic microlaser empowers it to achieve enantiomeric excess determination of various unknown enantiomers by a single technique, a feat that is challenging to accomplish with other methods.

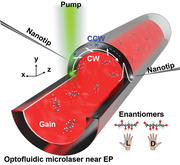

## 1 Introduction

Enantiomers (isomers with opposite chirality) exhibit similar physical and chemical properties due to their identical functional groups and compositions, yet showing different biological performances.^[^ ^1^, ^2^ ^]^ One enantiomer forms a powerful positive medicament, while the other may cause serious negative effects.^[^ ^3^, ^4^ ^]^ Consequently, enantiomeric excess (ee) determination (quantifying the purity of mixed enantiomers) is crucial during each step of the synthesis of chiral compounds, especially in the pharmaceutical industry.^[^ ^5^, ^6^, ^7^ ^]^ The ee of enantiomers is typically determined by both the chirality and concentration. Up until now, many methods including chiral high‐performance liquid chromatography,^[^ ^8^ ^]^ electrochemical method,^[^ ^9^ ^]^ and circularly polarized luminescence^[^ ^10^ ^]^ have been widely developed for chiral recognition. These methods require meticulous design of chiral stationary phases and chiral ligands for each compound, which is time‐consuming and decelerates the discovery pace. In recent decades, a range of sophisticated optical techniques have also been developed to determine the chirality of enantiomers in trace amounts, for example, photoionization,^[^ ^11^ ^]^ femtosecond,^[^ ^12^ ^]^ microwave,^[^ ^13^ ^]^ superchiral light spectroscopies,^[^ ^14^ ^]^ helical springs,^[^ ^15^ ^]^ circular dichroism,^[^ ^16^, ^17^, ^18^, ^19^, ^20^ ^]^ and cavity‐enhanced chiral polarimetry.^[^ ^21^, ^22^, ^23^ ^]^ However, such techniques need to be used in conjunction with other concentration measurement techniques for ee determination.^[^ ^24^ ^]^ Recently, an artificial intelligence algorithm was employed to predict the ee of enantiomers with unknown concentration from circular dichroism spectra.^[^ ^25^ ^]^ Nevertheless, it is still challenging to directly measure the ee of enantiomers under unknown concentration with a single technique.

Optofluidic microlasers, which integrate optical microcavities and gain materials in microfluidic environments, have emerged as state‐of‐the‐art integrated platforms for highly‐sensitive biological sensing.^[^ ^26^ ^]^ To date, optofluidic microlasers have been employed to distinguish subtle changes of biological processes, which are extracted from the variation in lasing signals of intensity, linewidth, wavelength, and polarization.^[^ ^27^, ^28^, ^29^, ^30^, ^31^, ^32^, ^33^, ^34^ ^]^ The whispering‐gallery mode (WGM) based optofluidic microlasers have attracted considerable research interest due to their high quality (*Q*) factors, enabling strong light–matter interactions.^[^ ^35^, ^36^, ^37^, ^38^, ^39^ ^]^ However, the WGM‐based optofluidic microlasers support both clockwise (CW) and counterclockwise (CCW) traveling‐wave modes with chiral symmetry.^[^ ^40^ ^]^ As a result, the chiroptical signals of enantiomers in the microfluidic are counteracted by the two counter propagating modes, which blocks its potential applications in detecting the chirality of biomolecules.^[^ ^41^ ^]^

In this work, WGM‐based optofluidic microlaser near the exceptional point (EP) was proposed to achieve simultaneous detection of enantiomers chirality and concentration. By introducing asymmetric backscattering, optofluidic microlasers operated at an EP to support unidirectional mode.^[^ ^42^, ^43^, ^44^, ^45^, ^46^, ^47^ ^]^ The polarization of the WGM lasing, that is, the relative intensity between transverse magnetic (TM) and transverse electric (TE) mode, can reflect the chirality of enantiomers, providing a novel capability for optofluidic microlaser. Simultaneously, the enantiomer concentration is revealed through the lasing wavelength, enabling one‐step ee determination without additional concentration measurements. Compared with the conventional optical rotation‐based polarimeter, the chiroptical response of the present approach was enhanced by ≈30‐fold, and the reagent consumption was reduced by three orders of magnitude. Furthermore, this approach was applied to detect all the eight essential amino acids (EAAs) enantiomers, indicating that it is applicable to all the enantiomers without elaborate design.

## 2 Results and Discussion

### 2.1 Design and Principles

**Figure 1:**
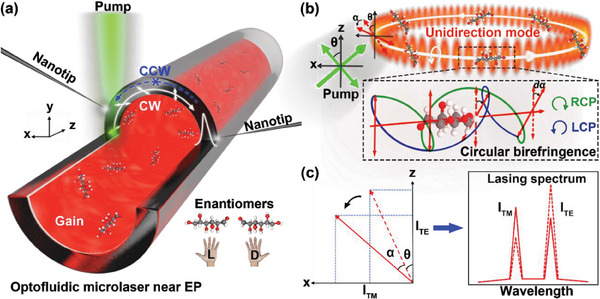
Optofluidic microlasers near EP for enantiomeric excess determination of enantiomers. a) Unidirectional lasing in the WGM based optofluidic microlaser when steered to an EP by two silica nanotips. b) WGM‐enhanced optical rotation effect. θ, the polarization angle of the excitation. α, the rotation angle of the lasing. Inset: the polarization deflection *d*α after the interaction with chiral molecules by circular birefringence. L/RCP, left/right circularly polarized light. c) The variation of intensity ratio of the TM and TE modes induced by the lasing polarization rotation.

The schematic of optofluidic microlasers near the EP is shown in **Figure** **1**. In our experiments, hollow optical fiber (HOF) integrates optical microcavity and microfluidic channel, allowing the strong interaction between light field and enantiomers, as well as enabling the injection of analytes via the capillary action. To avoid chiroptical signals of enantiomer being counteracted by the two counter propagating modes (CW and CCW modes), the optofluidic microlaser was steered to near an EP by using two silica nanotips as scatterers (see Figure S1, Supporting Information), which allowed the lasing to propagate only in the CW direction (Figure 1a).

The initial polarization of the unidirectional mode lasing (the dotted red arrow) was determined by the polarization of the pump source (the solid green arrow, top, Figure 1b), which was adjusted by a quarter wave plate and a linear polarizer (*P*_1_) (see Figure S1, Supporting Information). The angle between polarization direction of the *P*_1_ and the *Z*‐axis was defined as θ, which was zero when *P*_1_ was set along the *Z*‐axis direction. The unidirectional mode lasing was confined in the high‐*Q* WGM resonator to interact with chiral molecules for long periods of time, resulting in rotation of the lasing polarization (solid red arrow). The rotation of the polarization is attributed to the circular birefringence of chiral molecules,^[^ ^48^ ^]^ which produces a phase difference between the left and right circularly polarized light (L/RCP, bottom, Figure 1b). The rotation of the polarization leads to a change of relative intensity of TM and TE mode in the lasing spectrum (Figure 1c). Thus, the chirality of enantiomers can be detected by analyzing TM and TE modes of the lasing spectra.

### 2.2 Characterization

In the experiment, the HOF with an outer diameter of 140 µm and a wall‐thickness of 20 µm was used. Owing to the natural circular cross‐section, the WGM of the HOF has a *Q* factor of 5.75 × 10^5^ (see Figure S2, Supporting Information). A high *Q* factor implies a long photon lifetime in the optical cavity and ensures strong interaction between the light field and the materials, which enables high sensitivity and low lasing threshold. It is noted that only a micro volume of reagent (≈400 nL) was needed for one test owing to the miniature size of the HOF (see Figure S3, Supporting Information).

**Figure 2:**
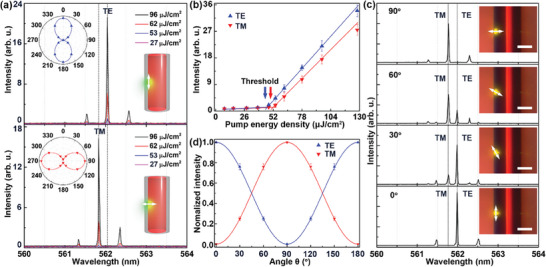
Emission characteristics of the optofluidic microlaser. a) The emission spectra of the optofluidic microlaser excited by two orthogonal polarization direction at different pump energy densities. Insets: the polar plots of the optofluidic laser polarization (left) and the excitation spot of pump laser focused on the edge of the WGM cavity (right). The pump polarization is indicated with white arrows. b) The evolution of laser emission intensities with the pump energy density. c) The lasing spectra varied with polarization angle of the pump laser at a fixed excitation energy density of 96 µJ cm^−2^. Insets: Bright‐field images of the optofluidic microlaser under optical pumping. Scale bar, 70 µm. d) Normalized laser intensity of TE and TM modes as a function of the polarization angle of the pump laser. Triangles, experimental data. Solid lines, fitting results.

**Figure** **2** shows the emission characteristics of the optofluidic microlaser. The evolution of emission spectra at different pump energy densities are shown in Figure 2a. The pump laser was focused on the edge of the WGM cavity with two orthogonal polarization directions (parallel and perpendicular to the axis of HOF, insets of Figure 2a). The white arrows indicate the polarization of the pump laser. When the optofluidic microlaser was excited by the pump laser with parallel polarization direction ( θ= 0°), the polarization of the emission was parallel to the HOF with a dominant peak at about 562.05 nm (TE mode, top, Figure 2a). The laser excitation with vertical polarization ( θ= 90°) generated polarized emission perpendicular to the HOF with a dominant peak at about 561.83 nm (TM mode, bottom, Figure 2a). The polarization was confirmed by a linear polarizer (*P*_2_) (see Figure S1, Supporting Information), as illustrated in the polar coordinate insets of Figure 2a. The emission intensities increased nonlinearly with the pump energy, achieving the lasing thresholds of about 48 and 53 µJ cm^−2^ for TE and TM modes, respectively (Figure 2b). Note that, the slight difference between the threshold of TE and TM modes was attributed to the difference in the coupling efficiency of the parallel and perpendicular pump laser.

To investigate the relationship of polarization between laser emission and pump source, we rotated the polarization angle of the pump source and recorded the lasing spectra with a step of 30°. Figure 2c presents a sequence of lasing spectra varied with the polarization angle of the pump source ( θ= 0°, 30°, 60°, and 90°) at a fixed pump energy density of about 96 µJ cm^−2^. Figure 2d shows the normalized intensity of TE and TM modes as a function of the polarization angle of the pump source. To account for the relation between the lasing modes and the polarization of the pump source, the dye molecule was treated as an induced dipole moment ($\vec{p}$), of which the direction was in accordance with the polarization of the pump source (see Figure S4, Supporting Information). The electric fields of emission generated by induced dipole moment can be expressed as $E_{\mathrm{em}}\propto K\times \left(K\times \vec{p}\right)$,^[^ ^28^, ^49^ ^]^ where *K* is the direction vector. In the WGM microcavity, only the photons satisfying the phase‐matching condition can be confined in the resonator to form lasing. In this case, the angle between *K* and $\vec{p}$ can be approximated to 90°, resulting in $E_{\mathrm{em}}\propto \vec{p}$. Therefore, the emission intensity of TE and TM components could be expressed as

(1) $$I_{\mathrm{TE}}\propto {\left|\vec{p}\right|}^{2}\cos^{2}\theta$$

(2) $$I_{\mathrm{TM}}\propto {\left|\vec{p}\right|}^{2}\sin^{2}\theta$$

which agrees well with the experimental results.

### 2.3 Steering the Optofluidic Microlaser to the EP

**Figure 3:**
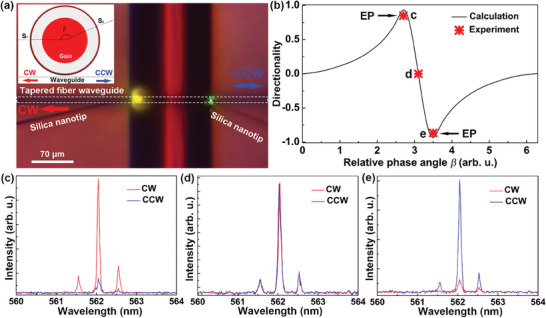
Scatterer‐induced optofluidic microlaser with unidirectional WGM mode near EP. a) Bright‐field image of tapered fiber‐HOF system. Two silica nanotips and tapered fiber (white dotted line) are used as scatterers and to couple out the CW/CCW lasing, respectively. Insets: schematic diagram of the cross‐section of the system. b) The directionality as a function of the relative phase angle *β* between two scatterers. c–e) Lasing spectra of the optofluidic microlaser coupled out by the tapered fiber at different position of two nanotips. Different directionalities are indicated: c) +0.8; d) 0; and e) −0.8.

To avoid the chiroptical signals of matters in the microfluidic being counteracted by CW and CCW propagating modes, the WGM‐based optofluidic microlaser was steered to an EP to break symmetry of microcavity and thus achieve lasing with unidirectional mode. In our experiment, EP‐broken symmetry was achieved by using two silica nanotips as the scatterers around the boundary of the HOF. The relative positions (i.e., relative phase angle *β*) of the nanotips were controlled by 3D control platform, and the direction of the lasing mode (CW or CCW) can be measured by coupling with the tapered fiber waveguide to a spectrometer (see **Figure** **3**a and Figure S1, Supporting Information).

In the slowly varying envelope approximation, the system can be modeled in the time domain with a Schrödinger‐like equation

(3) $$i\frac{d}{dt}\Psi =H\Psi$$

where Ψ is the complex‐valued 2D vector consisting of the field amplitudes of the CW and CCW propagating wave, which corresponds to the *e* ^±*im*φ^ angular dependence in real space; the positive integer *m* is the angular mode number; φis the relative phase angle of the scatterers. The corresponding effective Hamiltonian can be expressed as^[^ ^50^, ^51^, ^52^, ^53^ ^]^

(4) $$H=\left(\begin{array}{cc} \Omega & S_{1}+S_{2}e^{-i2m\beta }\\ S_{1}+S_{2}e^{i2m\beta } & \Omega \end{array}\right)$$

which is non‐Hermitian (see S5, Supporting Information). The diagonal element Ω is the complex mode frequency of the degenerate lossy WGM resonance. The complex valued off‐diagonal elements are the backscattering coefficient describing the scattering from the CCW (CW) to the CW (CCW) mode, where *S*_1_ and *S*_2_ are dependent on the size of the two scatterers, respectively. Thus, the ratio of CW and CCW modes can be varied by changing the backscattering coefficients of the Hamiltonian Equation (4) that are determined by the size and relative phase angle *β* of the two scatterers. To achieve EP, either of the backscattering coefficients is equal to zero to support a unidirectional mode, in which there is only backscattering from the CCW (CW) mode to the CW (CCW) mode.

The EP‐induced unidirectional mode was investigated theoretically and experimentally through the finite‐element analysis and tapered fiber coupling, respectively. Theoretically, we simulated the electric field distribution of the microcavity and utilized directionality to characterize the unidirectional mode. The directionality, *D* = (*I*_CW_ − *I*_CCW_)/(*I*_CW_ + *I*_CCW_), was defined as the coupled power distribution between the CCW and CW modes, and can be obtained from the integration of coupling light at the left (*I*_CW_) and right side (*I*_CCW_). According to the Equation (4), the directionality *D* was varied periodically by changing the relative phase angle *β* of the two scatterers at a fixed size. Figure S5 (Supporting Information) illustrates the intracavity field pattern for the cases *D* = ±1 and 0. The directionality *D* as a function of the relative phase angle *β* between the scatterers in one periodic obtained from the numerical simulation is summarized in Figure 3b, where the transition from bidirectional states to unidirectional at EPs are clearly seen. Experimentally, the high directionality unidirectional lasing (*D* = ±0.8) and bidirectional lasing (*D* = 0) were observed by precisely tuning the relative positions between the two silica nanotips. The recorded spectra from the fiber optical spectrometers are shown in Figure 3c–e, which correspond to the directionality values of +0.8, 0, and − 0.8 respectively. In the cases of *D* = ±0.8, the WGM based optofluidic microlaser could be approximated as operating near an EP with unidirectional laser emission, enabling high signal‐to‐noise in chirality recognition.

### 2.4 Discrimination of Chirality and ee

To explore the ability of optofluidic microlaser for chiral recognition and ee determination, the optofluidic microlaser was steered to an EP with lasing propagated only in the CW direction. Glucose with different chirality (l‐ and d‐glucose) was chose as a proof‐of‐concept. Eight essential amino acids were used to demonstrate the universality of the proposed technique.

**Figure 4:**
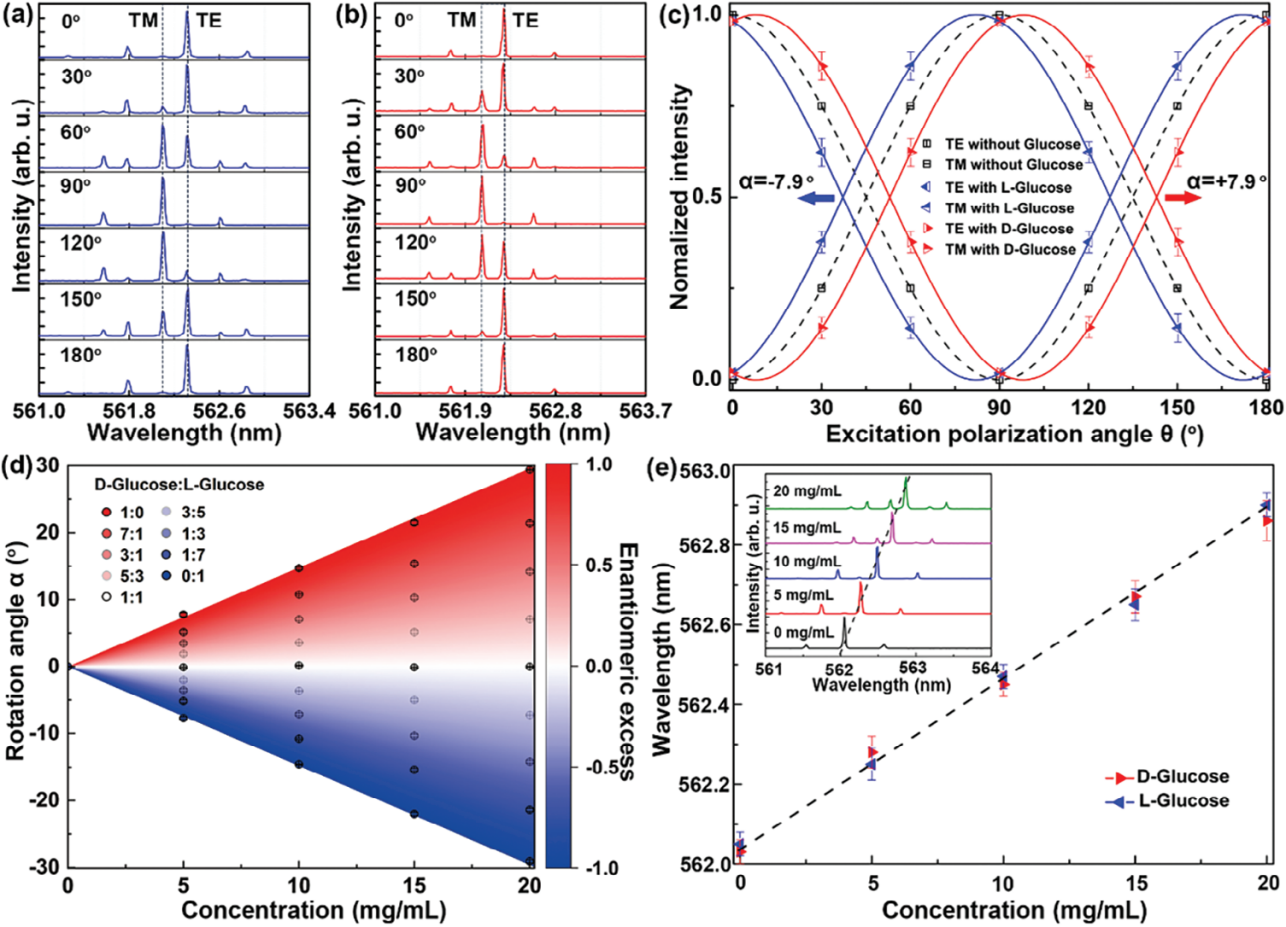
Chirality and purity detection of glucose molecules by the optofluidic microlaser near EP. Lasing spectra of the optofluidic microlaser with 5 mg mL^−1^ of a) l‐glucose and b) d‐glucose at different polarization directions of pump laser. c) The normalized intensity of TE and TM modes of optofluidic microlaser with l‐glucose, d‐glucose, and without glucose as a function of the excitation polarization angle. d) Rotation angleα and enantiomeric excess of l‐ and d‐glucose at various concentration. Circles, experimental data. Color map, fitting results. e) Lasing wavelength shift at different concentration of l‐ and d‐glucose. Inset: the evolution of spectra with the concentration of d‐glucose at the same excitation polarization angle of 0°.

Turning the polarization angle of the pump source, the lasing spectra were recorded with a step of 30° at a fixed excitation energy density of 96 µJ cm^−2^ (same as section 2.1). **Figure** **4**a,b presents a sequence of the excitation polarization angle dependent spectra of optofluidic microlaser containing 5 mg mL^−1^ solution of l‐glucose and d‐glucose, respectively. The normalized intensity of TE and TM mode of optofluidic microlaser with l‐glucose, d‐glucose, and without glucose as a function of the excitation polarization angle are summarized in Figure 4c. Compared to the optofluidic microlaser without glucose (black dashed lines), the excitation polarization angle dependent intensity curves of TE and TM mode with l‐glucose (blue lines) shifted an angle α of −7.9 degrees, while the intensity curve of TE and TM modes with d‐glucose (red lines) shifted by almost the same angle but in the opposite direction (Figure 4c). These results indicated that the variation of the relative intensity between the TM and TE mode with the excitation polarization angle no longer exactly followed the description in Equations (1) and (2), but the polarization was rotated at an angle α by the chiral molecules in the resonator. Therefore, the emission intensity of TE and TM components of optofluidic microlaser with chiral molecules can be expressed as

(5) $$I_{\mathrm{TE}}\propto {\left|\vec{p}\right|}^{2}\cos^{2}\left(\theta +\alpha \right)$$

(6) $$I_{\mathrm{TM}}\propto {\left|\vec{p}\right|}^{2}\sin^{2}\left(\theta +\alpha \right)$$

where *α* is the rotated angle induced by chiral molecules and the sign is the chirality of the molecule. A positive rotation angle indicated dextrorotatory chirality (d‐glucose), while a negative angle indicated levorotatory chirality (l‐glucose). Thus, the chirality of molecules can be extracted from the variation of the relative intensity between the TM and TE mode with the excitation polarization angle. By contrast, in the control experiment without asymmetric backscattering, the existence of chiral molecules did not change the polarization angle of laser emission (i.e., *α* = 0), confirming the chiroptical signals of glucose being counteracted in the symmetry system (see Figure S6, Supporting Information).

The ee is an important parameter for enantiomer purification. Here, we defined the ee of glucose as ee = (*C*_D_ − *C*_L_)/(*C*_D_ + *C*_L_), where *C*_L_ and *C*_D_ were the concentration of l‐ and d‐glucose, respectively. The ee mapping of glucose against rotation angle *α* and the concentration *C* was investigated by mixing the l‐ and d‐glucose in different proportions and gradually increasing the concentration. Figure 4d shows the calibration curve of glucose ee determination with the dependence of $\mathrm{ee}=\frac{\alpha }{1.49C}$. As a result, the ee of glucose could be obtained by measuring chirality at a given concentrations. Note that, for the pure l‐/d‐glucose (ee = ±1), the *α* increased with the concentration at a slope of ± 1.49° per mg mL^−1^, which was the maximum chiroptical signal response of glucose in the system. Compared to conventional optical rotation‐based polarimeter (≈0.049° per mg mL^−1^, volume of reagent ≈1.2 mL), the chiroptical signal response of the optofluidic microlaser approach (≈1.49° per mg mL^−1^, volume of reagent ≈400 nL) was improved by ≈30‐fold and the required reagent volume was reduced by three orders of magnitude (see Figure S7, Supporting Information). Additionally, the limit of detection (LOD) of the system for chirality of pure glucoses was calculated to be 0.01 mg mL^−1^, according to the formula LOD = 3σ_D_/*b*,^[^ ^9^ ^]^ where *b* is the chiroptical signal response of pure glucose, and σ_D_ is the standard deviation of the rotation angle (see Figure S8, Supporting Information).

More importantly, one of the another intriguing features of the optofluidic microlaser is that the lasing wavelength is sensitive to the concentration‐induced variation of refractive index, enabling the concentration detection of enantiomers. In the WGM microlaser, the lasing wavelength is expressed as λ_*m*_ = *n*π*D*/*m*, where λ_*m*_ is the lasing wavelength, *n* is the effective refractive index of the cavity, *D* is diameter of WGM cavity, and *m* is the angular mode number. Generally, the refractive index of the reagent increases with the concentration, which results in red‐shift of the lasing peaks.^[^ ^54^ ^]^ The inset of Figure 4e presents a sequence of spectra evolution with the concentration of d‐glucose at fixed the excitation polarization angle of 0°. The lasing peaks show a red‐shift of about 0.82 nm as the concentration increased from 0 to 20 mg mL^−1^. The lasing wavelength as a function of the concentration for both l‐and d‐glucose are summarized in Figure 4e, where the linear relationship was expressed as λ_*m*_ = 0.04*C* + 560.82. The LOD of the system for the concentration of glucoses can be calculated to be 0.07 mg mL^−1^ (see Figure S8, Supporting Information). Therefore, the concentration information of enantiomer can be simultaneously revealed through the lasing wavelength, enabling ee determination of unknown concentration enantiomers without additional concentration measurements, which was difficult to achieve within any other methods. Finally, a single‐blind test was also conducted on the proposed technique to detect the ee of glucose with an unknown concentration (above LOD), demonstrating a significantly higher detection accuracy of up to 98.6% compared to the data‐based prediction approach^[^ ^25^ ^]^ (see Figure S9, Supporting Information).

**Figure 5:**
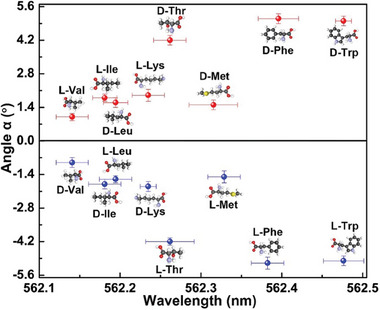
Chirality detection of EAAs enantiomers. The rotation angle and emission wavelength of optofluidic microlaser near EP are obtained from 5 mg mL^−1^ solution.

Finally, the universality of this approach were evaluated by applying it for the chiral detection of all the EAAs enantiomers, including Val, Ile, Leu, Lys, Thr, Met, Phe, and Trp. The lasing characteristics were recorded from optofluidic microlaser near EP, containing 5 mg mL^−1^ solution of different EAAs (see Figure S10, Supporting Information). The addition of different EAAs resulted in significantly change in the rotation angle and lasing wavelength (**Figure** **5**). The EAAs enantiomers of the same species caused almost the same lasing wavelength but opposite rotation angle, while the EAAs of different species showed different lasing wavelength. Thus, the ee determination of all the eight EAAs enantiomers could be achieved by optofluidic microlaser near EP, indicating that this method is applicable to all types of chiral chemicals without the specific design for different molecules.

## 3 Conclusion

A WGM‐based optofluidic microlaser near the EP is proposed to achieve one‐step ee determination of unknown concentration enantiomers. Breaking symmetry by EP, the chirality of enantiomers can be extracted by analyzing TM and TE modes of the WGM lasing spectra. Featuring the sensitivity enhancement and the miniature size of the WGM‐resonator, the chiroptical response of the present approach is ≈30‐fold higher than that of the conventional optical rotation‐based polarimeter, and the reagent consumption is reduced by three orders of magnitude. More importantly, the measured lasing spectra can reveal the concentration of enantiomers through the lasing wavelength, enabling ee determination without extra concentration measurements. Moreover, the universality of this approach is demonstrated by detecting the eight EEAs enantiomers. The present work provides an integrated strategy for the detection of ee of unknown concentration enantiomers by using a single technique. Due to the high sensitivity, multifunction, and universality, the optofluidic microlaser near EP can be applicable to all types of chiral chemicals without the specific design and greatly extends the applications in pharmacochemistry.

## 4 Experimental Section

### Sample Preparation

The enantiomers d‐glucose, l‐glucose, d‐Val, l‐Val, d‐Ile, l‐Ile, d‐Leu, l‐Leu, d‐Lys, l‐Lys, d‐Thr, l‐Thr, d‐Met, l‐Met, d‐Phe, l‐Phe, d‐Trp, l‐Trp, and the typical laser dye rhodamine 6G (R6G) were purchased from Aladdin Biotech, China. The HOFs were purchased from Polymicro Technologies, USA. The reagent was prepared by dissolving enantiomers in a 1:1 mixture of alcohol and deionized water with R6G of 2 mg mL^−1^. Then, the reagent was filled into the hollow core of the HOF by capillary action and the HOF was excited by the pump source to demonstrate lasing and sensing.

### Fabrication of the Tapered Fiber Waveguide and Silica Nanotips

The coupling tapered fiber waveguide was fabricated by pulling a standard single‐mode optical fiber under oxyhydrogen flame until the waist diameter was about 1 µm for high coupling efficiency. The silica nanotips used as scatterers in the experiments were fabricated by breaking a tapered fiber that was further etched by hydrofluoric acid. The nanotip had a cone shape, with diameter varying from a hundred to several hundreds of nanometers.

### Optical Measurement

A *Q*‐switched second‐harmonic Nd:YAG pulsed laser (MINILITE II, 532 nm, 5–7 ns pulse duration, 15 Hz repetition rate) was employed as the pump source. The emission spectra were collected by a spectrometer with a grating of 2400 g mm^−1^ (0.01 nm resolution, SpectraPro HRS‐500, PI). The direction of a lasing mode (CW and CCW) was measured by coupling the tapered fiber waveguides with HOF and then splicing with fiber optical spectrometers (0.05 nm resolution, Ocean Optics HR4000). The commercial polarimeter (P‐2000, JASCO) was used for the comparative experiments.

## Conflict of Interest

The authors declare no conflict of interest.

## Author Contributions

Conceptualization, Z.X., and T.Z.; methodology, Z.X., X.Y., and Y.Y.; theoretical and numerical calculations, X.W., X.Y., and Z.X.; validation, Y.Y., and X.Y.; formal analysis, X.W., and Z.Z.; investigation, Z.X., and T.Z.; writing‐original draft preparation, Z.X.; writing, review and editing, Y.Y., X.Y., and T.Z.; supervision, T.Z.; project administration, T.Z.; funding acquisition, T.Z., and Y.Y. All authors have read and agreed to the published version of the manuscript.

## Supporting information

Supporting Information

## Data Availability

The data that support the findings of this study are available from the corresponding author upon reasonable request.
