# Supplementary material for: Determination of Enantiomeric Excess by Optofluidic Microlaser near Exceptional Point
Source: Adv Sci (Weinh). 2023 Dec 10;11(7):2308362. doi: 10.1002/advs.202308362 (PMC10870016; doi:10.1002/advs.202308362)
Supplement: Supplementary file 1 — Supporting Information [file ADVS-11-2308362-s001.pdf]

ADVANCED SCIENCE
Open Access

Supporting Information

for *Adv. Sci.*, DOI 10.1002/advs.202308362

Determination of Enantiomeric Excess by Optofluidic Microlaser near Exceptional Point

Zhiyang Xu, Yinzhou Yan, Xingyuan Wang, Xiaolei Wang, Zhixiang Zhou, Xi Yang and Tianrui Zhai**

Supplementary Information

Determination of Enantiomeric Excess by Optofluidic Microlaser near Exceptional Point

Zhiyang Xu[1,2], Yinzhou Yan[2], Xingyuan Wang[3], Xiaolei Wang[1], Zhixiang Zhou[4], Xi Yang[5]* and Tianrui Zhai[1]*

[1]*Department of Physics and Optoelectronic Engineering, Faculty of Science, Beijing University of Technology, Beijing 100124, China*

[2]*Institute of Laser Engineering, Faculty of Materials and Manufacturing, Beijing University of Technology, Beijing 100124, China*

[3]*College of Mathematics and Physics, Beijing University of Chemical Technology, Beijing 100029, China*

[4]*Faculty of Environment and Life, Beijing University of Technology, Beijing, 100124, China*

[5]*State Key Laboratory for Mesoscopic Physics and School of Physics, Peking University, Beijing 100871, China*

*Correspondence: Tianrui Zhai (trzhai@bjut.edu.cn)

CONTENTS

S1. Experimental setup

S2. Parameters of the hollow optical fiber

S3. Consumption of reagents

S4. Fluorescence characteristics of rhodamine 6G dyes

S5. Theoretical model of optofluidic microlaser near EP

S6. Controlled experiment of the optofluidic microlaser not near the EP

S7. Experiment of D-glucose detection by conventional optical rotation polarimeter

S8. Stability of the optofluidic microlaser near EP

S9. Single-blind test on the ee of glucose with an unknown concentration

S10. The lasing characteristics of the eight EAAs and their enantiomers near EP

S1. Experimental setup

A homemade microscopic system is employed to characterize the lasing properties of optofluidic microlaser and determine the ee of enantiomers, as shown in Figure S1. The polarization of the pump source was manipulated by a quarter wave plate and a linear polarizer (P_1) in the excitation optical path. The polarization properties of emission were confirmed by another linear polarizer (P_2) in the measurement optical path.

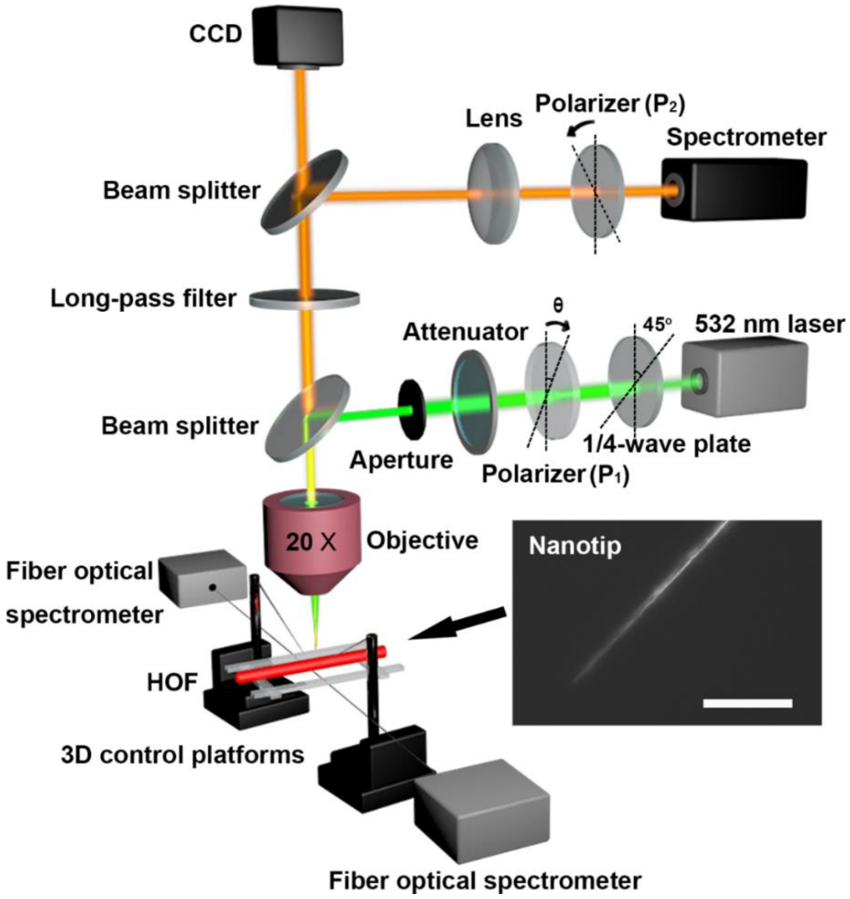

Figure S1. The schematic of the experimental setup. Insets: bright-field image of the silica nanotip as scatter. Scale bar, 10 μm.

S2. Parameters of the hollow optical fiber

The hollow optical fiber (HOF) was an ideal design for the optofluidic microlaser, which allowed the reagents with gain medium to flow through the hollow core via the capillary action to interact with the evanescent field of WGMs. In the experiment, the commercial HOF with an outer diameter of 140 μm and a thickness of 20 μm was employed, as shown in Fig. S2a. To determine the *Q* factor of the HOF cavity, the WGMs of the HOF were excited by a tunable laser at a wavelength band of 635 nm via the evanescent coupling with the fiber taper. Then, the transmission light of the fiber taper was collected by a low-noise photodetector and analyzed by an oscilloscope. A typical transmission spectrum is shown in Fig. S2b. The *Q* factor of the HOF cavity was measured to be 5.75×10^5.

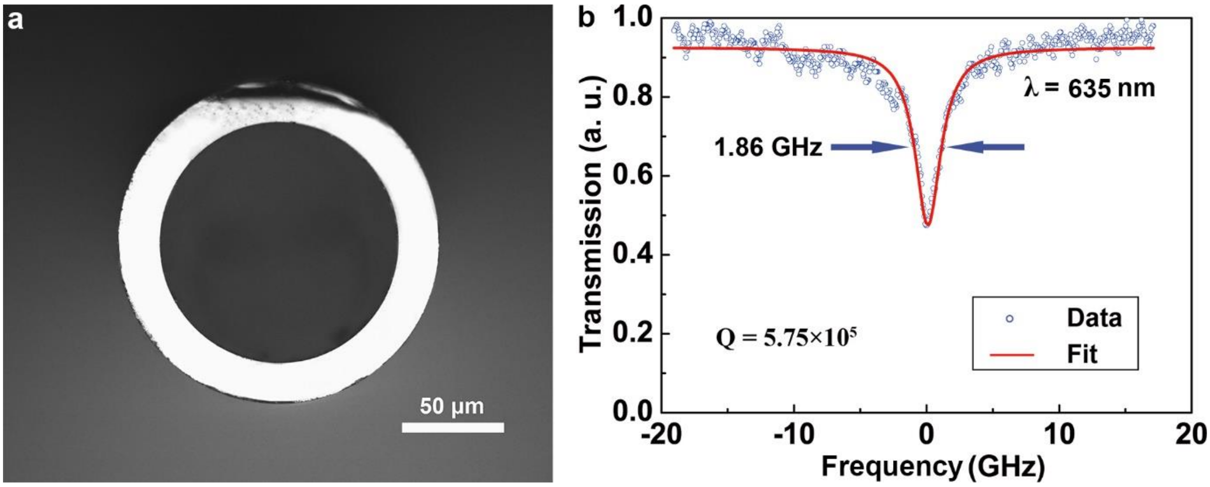

Figure S2. (a) The SEM image of the cross-section of the HOF, showing an outer diameter of 140 μm with a thickness of 20 μm. (b) Representative *Q* factor of HOF cavity. Here, a fiber taper was used to couple the probe light from a tunable laser in 635 nm wavelength band into the WGMs of the microcavity and to collect the output as transmission spectra.

S3. Consumption of reagents

The enantiomer reagents with gain medium flowed through the hollow core of HOF via the capillary action. Owing to the miniature size of the HOF, only a small volume of reagent (~400 nL) was needed for one test.

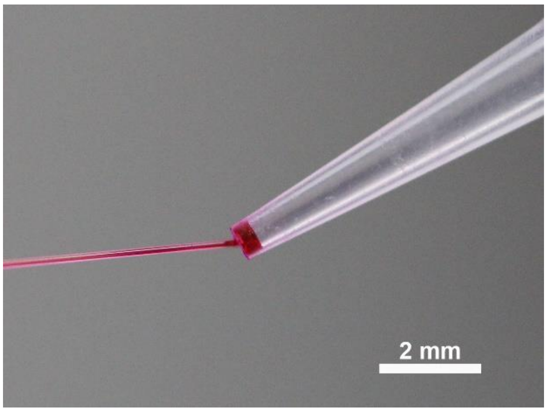

Figure S3. The image for reagents absorbing into the HOF via capillary action.

S4. Fluorescence characteristics of rhodamine 6G dyes

In this work, the typical laser dye rhodamine 6G (R6G) was used as the gain medium for the optofluidic microlaser. The laser dye is racemic without chirality, and thus treated as an induced dipole moment [1]. In this case, the polarization of their fluorescence is closely related to the polarization of excitation light, which is due to the significant influence of excitation light on the oscillatory mode of the electron cloud of the fluorescent molecule. Therefore, the emitted fluorescence will exhibit a similar polarization with excitation light [2]. Figure S4a shows the normalized absorbance and photoluminescence (PL) spectra of the R6G solution. The polar plot of PL polarization is shown in Fig. S4b, which was identical to the polarization field of excited light with a polarizability of about 25%. Thus, the R6G molecule can be treated as an induced dipole moment.

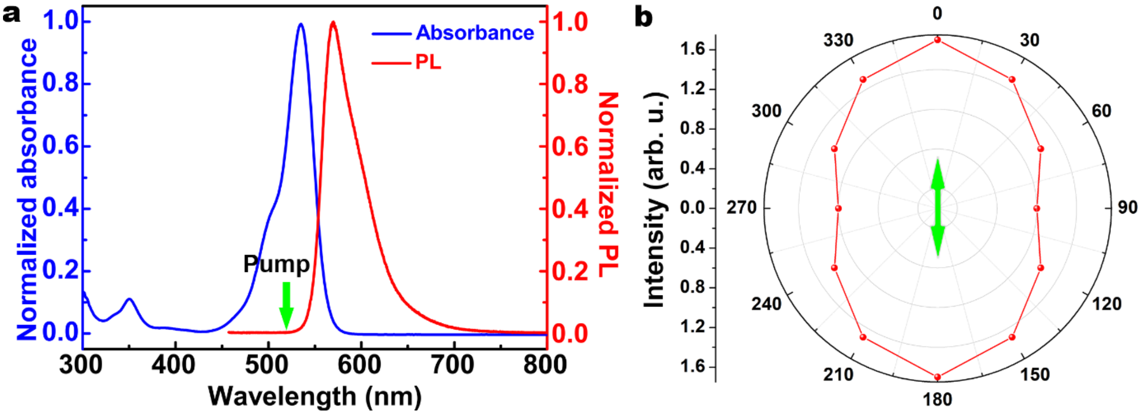

Figure S4. (a) Normalized absorbance and photoluminescence (PL) spectra of rhodamine 6G. The wavelength of the pump is indicated by the green arrow. (b) The polar plots of the PL polarization. The green arrow shows the polarization of the excited light.

S5. Theoretical model of optofluidic microlaser near EP

Starting from the HOF cavity without scatterers, a pair of degenerate standing-wave modes of an isolated WGM microcavity with a frequency Ω and azimuthal mode number *m*, the corresponding effective Hamiltonian of Eq. (1) of the main text can be expressed as

$$\mathrm{H}_0 = \begin{pmatrix} \Omega & 0 \\ 0 & \Omega \end{pmatrix}, \tag{S1}$$

which can be approximated as Hermitian system. Once a single scatterer was introduced the WGM cavity at position of $\varphi = 0$ with the coupling coefficient of S_1, in the traveling-wave basis of CCW and CW modes, the coupling Hamiltonian can be expressed as

$$\mathrm{H}_1 = \begin{pmatrix} 0 & S_1 \\ S_1 & 0 \end{pmatrix}. \tag{S2}$$

A second scatterer at the position $\varphi = \beta$ with the coupling coefficient of S_2 can be treated in a similar way, where the transformation to the traveling-wave basis should include the relative phase angle β:

$$\mathrm{H}_2 = \begin{pmatrix} 0 & S_2 e^{-i2m\beta} \\ S_2 e^{i2m\beta} & 0 \end{pmatrix}. \tag{S3}$$

Thus, the total effective Hamiltonian in the traveling-wave basis can be expressed as

$$\mathrm{H} = \mathrm{H}_0 + \mathrm{H}_1 + \mathrm{H}_2 = \begin{pmatrix} \Omega & S_1 + S_2 e^{-i2m\beta} \\ S_1 + S_2 e^{i2m\beta} & \Omega \end{pmatrix}. \tag{S4}$$

While the diagonal elements describe the complex frequency, the off-diagonal terms give the backscattering coefficient. Specifically, the variations in the off-diagonal terms lead to differences in the proportion of CCW and CCW components within the system. Thus, when either of the backscattering coefficients is zero, the system operates at an EP, achieving a unidirectional mode.

Finite-element analysis was used to simulate the electric field distribution of this system. The directionality, $D=\left(I_{CW}-I_{CCW}\right)/\left(I_{CW}+I_{CCW}\right)$, was defined as the coupled power distribution between the CCW and CW modes and can be obtained from the integration of coupling light at the left (I_{CW}) and right side (I_{CCW}). The directionality of $D=\pm1$ and 0 were obtained by varying the relative phase angle between the scatterers, as shown in Figs. S5**a-c**. Note that, an imbalance between CW and CCW propagating modes inside the cavity leaded to destruction of interference waves. Consequently, the modes of the cavity were no longer standing waves but traveling copropagating waves with an indiscernible interference pattern, as shown in the insets of Figs. S5**a-c**, which was one of the characteristics of EP [3].

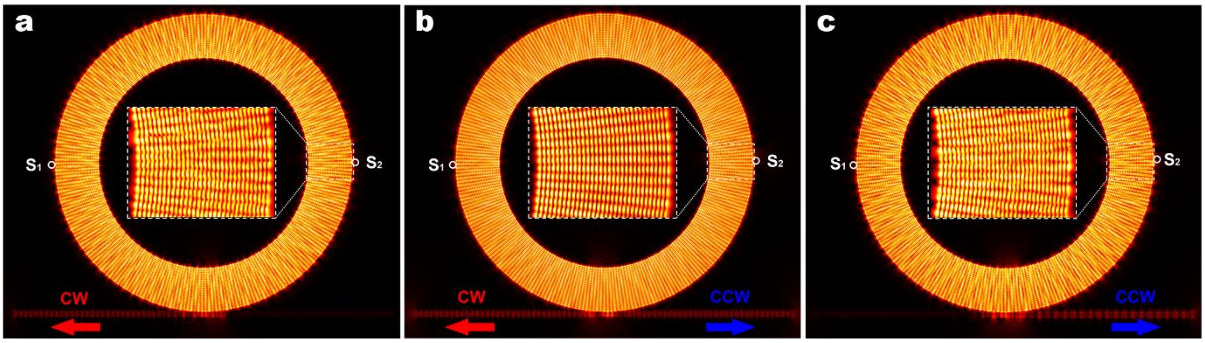

Figure S5. Results of finite element simulations at different relative phase angles β revealing the intracavity field patterns and output direction in the waveguides. β values are: (a) 3.1357 rad; (b) 3.1353 rad; and (c) 3.1349 rad. S_1 and S_2 denote the locations of the scatterers.

S6. Control experiment of the optofluidic microlaser without EP

A control experiment was performed by the optofluidic microlaser without introducing the asymmetric backscattering (without EP). The normalized intensity of TE and TM mode of optofluidic microlaser with L-glucose, D-glucose and without glucose as a function of the excitation polarization angle are illustrated in Fig. S6. It indicates that the lasing polarization has no significant change after the addition of L-glucose, D-glucose molecules. This result proved that the chiroptical signal of matters in the optofluidic microlaser was counteracted by the CW and CCW propagating modes. Therefore, the optofluidic microlaser must operate near EP for ee determination of enantiomers.

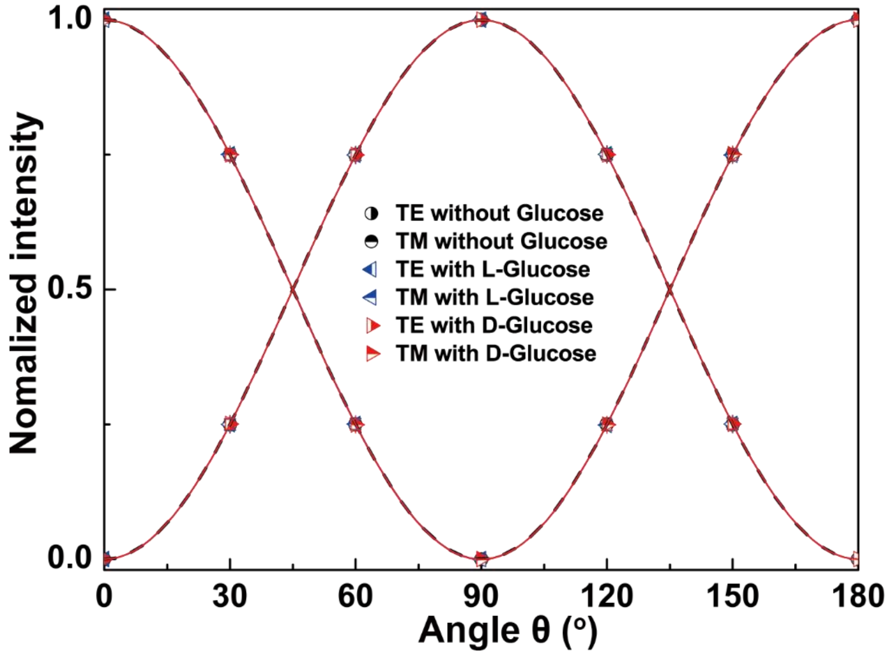

Figure S6. The normalized intensity of TE and TM mode of optofluidic microlaser (without EP) with L-glucose, D-glucose and without glucose as a function of the excitation polarization angle.

S7. Chiral detection of D-glucose via conventional optical rotation polarimeter

For a comparison, the chirality of pure D-glucose was determined by an optical rotation based commercial polarimeter (P-2000, JASCO). The optical tube length of the polarimeter is 10 mm and the consumption of reagent volume is 1.2 mL. Figure S7 shows the calibration curves of D-glucose obtained from commercial polarimeter and optofluidic microlaser. The sensitivity of commercial polarimeter for chiral detection is 0.049 degrees/mg mL^{-1}, while that of the optofluidic microlaser boosts to 1.49 degrees/mg mL^{-1}. Compared to the polarimeter, the sensitivity of optofluidic microlaser approach (~1.49 degrees/mg mL^{-1}, consumption of reagent ~400 nL) was improved by ~30-fold, and the consumption of reagent was reduced by three orders of magnitude.

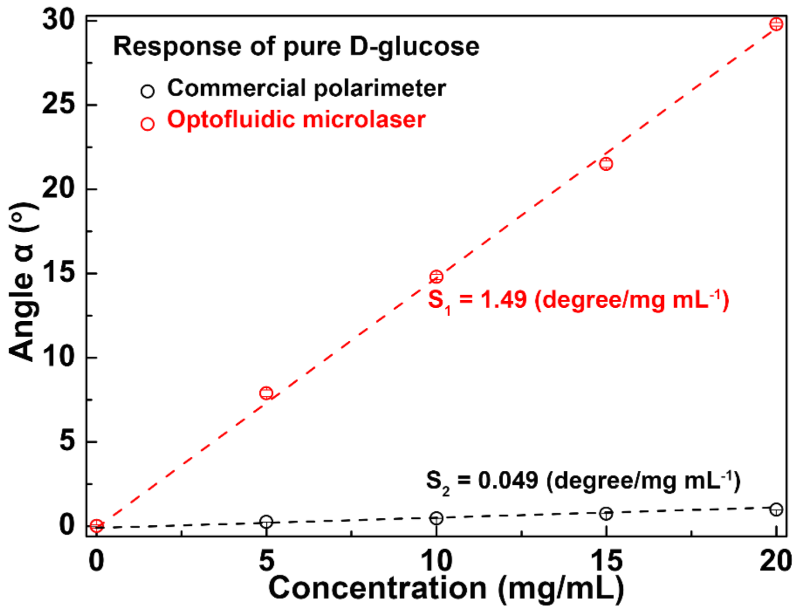

Figure S7. The rotation angle α as a function of the concentration of pure D-glucose measured by the commercial polarimeter (black) and optofluidic microlaser approach (red). Circles, experimental data. Solid lines, fitting results.

S8. Stability of the optofluidic microlaser near EP

To evaluate the stability of the optofluidic microlaser near EP, we conducted 40 tests on blank group without glucose. The system exhibited standard deviation of 0.005 degree and 0.001 nm in lasing polarization and wavelength, respectively.

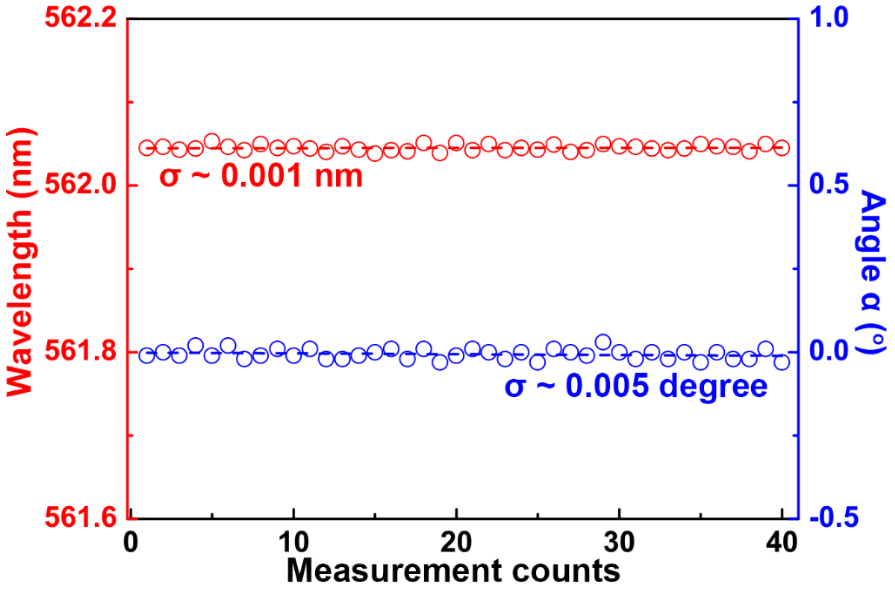

Figure S8. Stability of the lasing wavelength (red) and polarization (blue) of blank group without glucose. Circles, experimental data. Dashed lines, fitting results.

S9. Single-blind test on the ee of glucose with an unknown concentration

To assess the feasibility of the ee determination with unknown concentration, a single-blind test was conducted on the proposed technique. By directly measuring the wavelength and rotation angle of the laser spectrum from an unknown sample, both the concentration and chirality of the sample can be obtained, thereby enabling the calculation of the sample's ee by the formula $ee = \frac{\alpha}{1.49C}$. It is found that the measured ee varied linearly to the true ee values of glucose with a slope of 0.986. This indicates that the proposed method

can achieve ee determination of unknown concentration enantiomers with high accuracy.

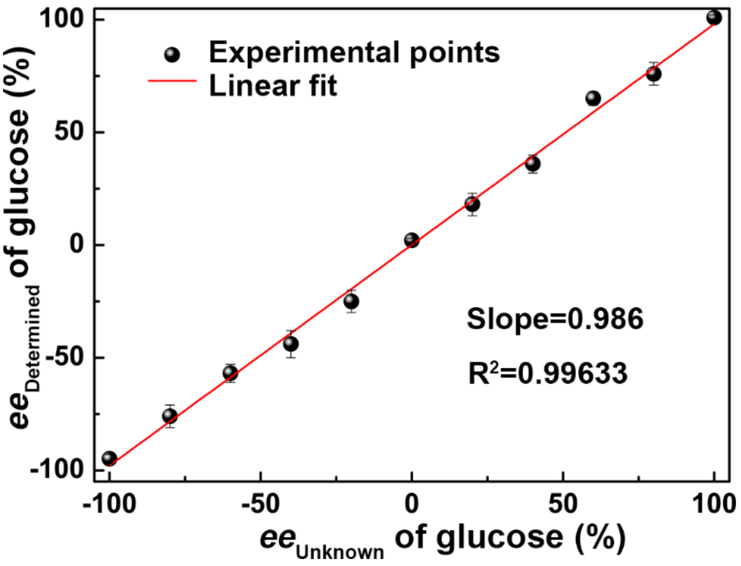

Figure S9. A single-blind test on the ee of glucose with unknown concentrations.

S10. The lasing characteristics of the eight EAAs and their enantiomers at EP

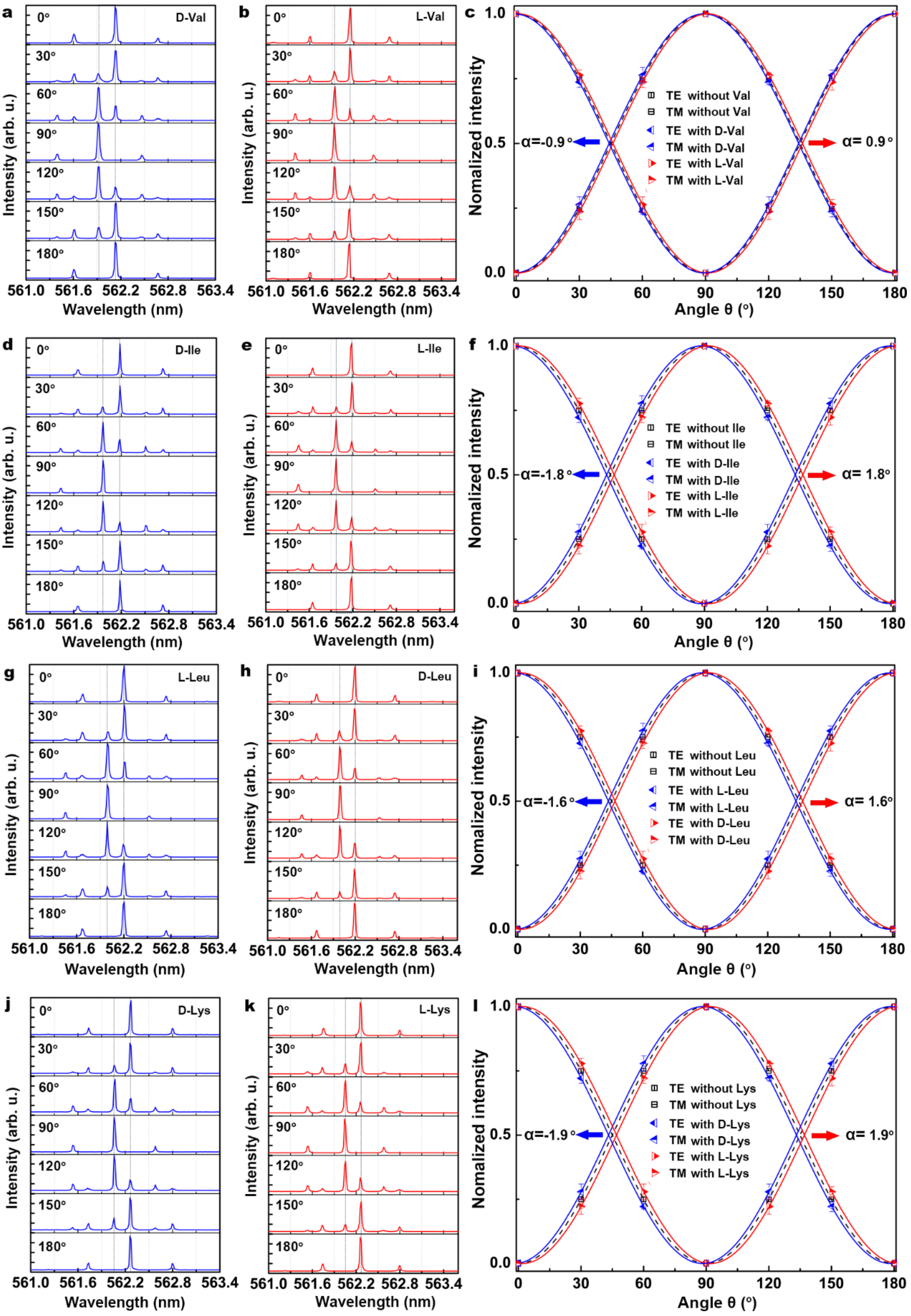
a
b
c
d
e
f
g
h
i
j
k
l
D-Val
L-Val
D-Ile
L-Ile
L-Leu
D-Leu
D-Lys
L-Lys
0°
30°
60°
90°
120°
150°
180°
Intensity (arb. u.)
Wavelength (nm)
561.0 561.6 562.2 562.8 563.4
Nomalized intensity
Angle θ (°)
0 30 60 90 120 150 180
0.0 0.5 1.0
TE without Val
TM without Val
TE with D-Val
TM with D-Val
TE with L-Val
TM with L-Val
α=-0.9°
α= 0.9°
TE without Ile
TM without Ile
TE with D-Ile
TM with D-Ile
TE with L-Ile
TM with L-Ile
α=-1.8°
α= 1.8°
TE without Leu
TM without Leu
TE with L-Leu
TM with L-Leu
TE with D-Leu
TM with D-Leu
α=-1.6°
α= 1.6°
TE without Lys
TM without Lys
TE with D-Lys
TM with D-Lys
TE with L-Lys
TM with L-Lys
α=-1.9°
α= 1.9°

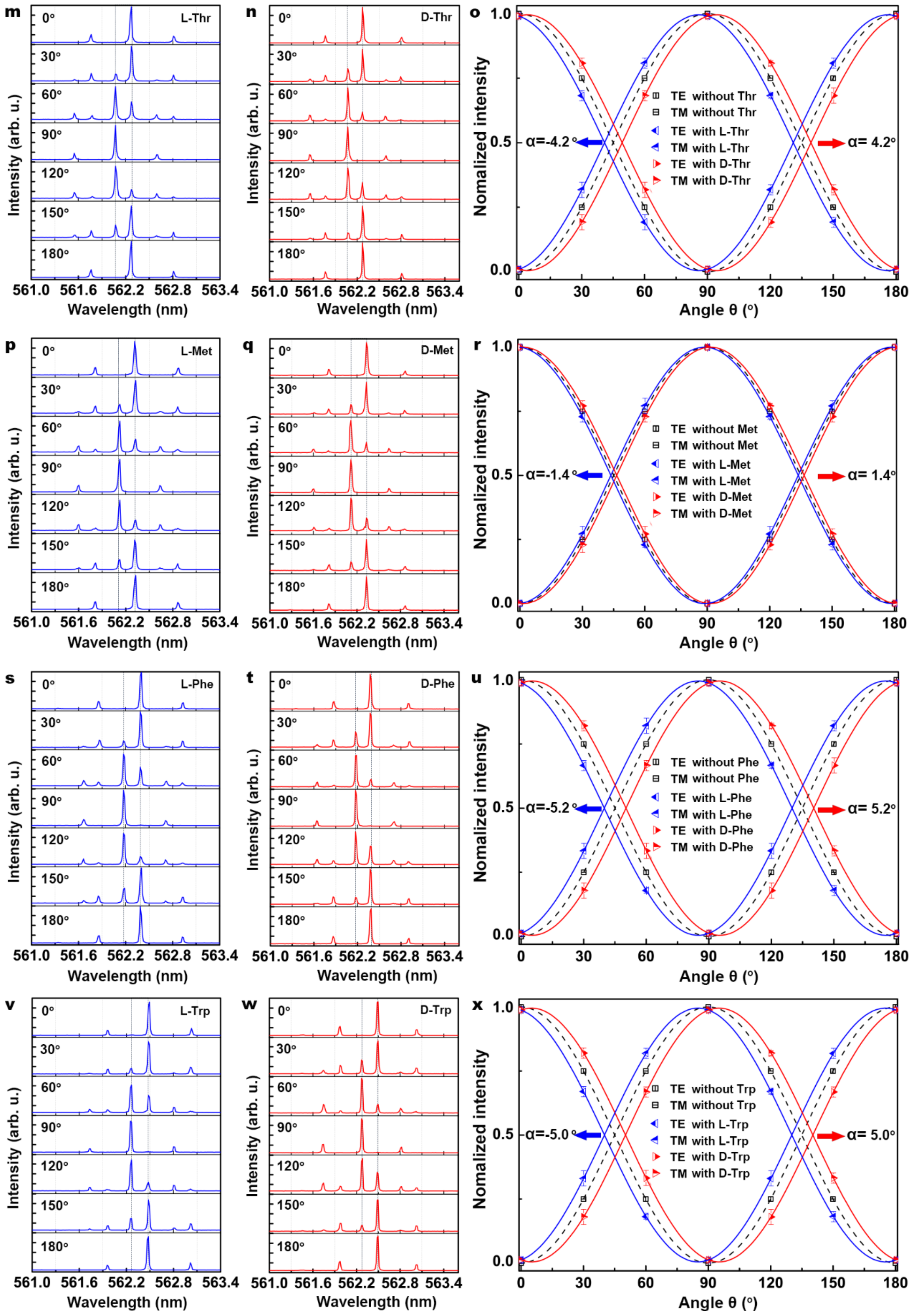

Figure S10. The excitation polarization angle dependent spectra and the normalized intensity of TE and

TM mode as a function of the excitation polarization angle from optofluidic microlaser at EP containing 5 mg mL^{-1} solution of (**a**)-(**c**) L- and D-Val, (**d**)-(**f**) L- and D-Ile, (**g**)-(**i**) L- and D-Leu, (**j**)-(**l**) L- and D-Lys, (**m**)-(**o**) L- and D-Thr, (**p**)-(**r**) L- and D- Met, (**s**)-(**u**) L- and D-Phe, and (**v**)-(**x**) L- and D-Trp.

The optofluidic microlaser near EP was also applied to measure the chirality of different amino acids enantiomers. We measured eight essential amino acids with the concentration of 5 mg mL^{-1}, including Val, Ile, Leu, Lys, Thr, Met, Phe and Trp. Figure S8 shows the excitation-polarization-dependent spectra and the normalized intensity of TE and TM mode as a function of the excitation polarization angle. It is obvious that the EAAs of the same species but in opposite configurations had almost the same emission wavelength, while their rotation angles are numerically equal and opposite in direction. For the EAAs of different species showed different emission wavelength as well as rotation angle. Thus, the determination of species and chirality of all the eight EAAs were achieved by optofluidic microlaser near EP.

Reference

1. Y. Zhang, W. Meng, H. Yang, Y. Chu, X. Pu, Demonstration of polarization mode selection and coupling efficiency of optofluidic ring resonator lasers. *Opt. Lett.* **2015**, 40, 5101-5104.

2. J. D. Jackson, Classical Electrodynamics (Wiley, **1962**).

3. B. Peng, S. Özdemir, M. Liertzer, W. Chen, J. Kramer, H. Yilmaz, J. Wiersig, S. Rotter, L. Yang, Chiral modes and directional lasing at exceptional points. *Proc. Natl. Acad. Sci.* **2016**,113, 6845-6850.
